# Environmental and occupational respiratory diseases – 1061. The change of eosinophil, serum level of ECP, and interleukin-5 during mycoplasma pneumoniae infection in atopic children

**DOI:** 10.1186/1939-4551-6-S1-P59

**Published:** 2013-04-23

**Authors:** Jae-Won Oh, Dong-Hoon Han, Ha-Baik Lee

**Affiliations:** 1 Pediatrics, Hanyang University, Seoul, South Korea

## Background

A number of studies have outlined mechanisms by which mycoplasma infection may promote allergic lung inflammation. In addition, there is increasing evidence from human studies suggesting that mycoplasma infection contribute to asthma exacerbations, and severity with the change of cytokines. The present study evaluated the change of serum levels of eosinophil count, eosinophil cationic protein, and interleukin-5 in atopic children with Mycoplasma pneumonia infection.

## Methods

We recruited 145 children including 45 atopic children with mycoplasma pneumonia (Group 1), 39 non-atopic children with mycoplasma pneumonia (Group 2), 35 children with viral pneumonia (group 3), 26 non-atopic children with viral pneumonia with mycoplasma infection (Group 4). The change of total eosinophil count, serum levels of interleukin (IL)-5, eosinophil cationic protein were measured at admission and at recovery for each group by using commercial ELISA.

## Results

The serum level of IL-5 at admission was increased at recovery in group 1 (114±51.1 pg/mL at admission, 143.2±68.4 pg/mL at recovery). However, Buserum eosinophil cationic protein concentrations were increased at clinical recovery compared to the mean serum concentration at admission (49.5 pg/mL at admission, 37.9 pg/mL at recovery in group 1; 38.2 pg/mL at admission, 27.8 pg/mL at recovery).

## Conclusions

The outcomes of the present study implied changes of eosinophil and its mediators during *Mycoplasma* infection may be associated with the mechanism by which the *Mycoplasma pneumoniae* contribute to the development of airway hypersensitivity.

